# Transgression and Gender

**DOI:** 10.1007/s11126-016-9454-y

**Published:** 2016-07-12

**Authors:** Konstantinos Tsirigotis

**Affiliations:** Department of Psychology, The Jan Kochanowski University in Kielce, Piotrków Trybunalski Branch, Słowackiego 114/118 str., 97-300 Piotrków Trybunalski, Poland

**Keywords:** Transgression, Gender, Femininity, Masculinity

## Abstract

Human activity is determined to a great extent by not only biological sex but also gender. The aim of the study was to examine relationships between transgression and types of gender. A population of 558 individuals (399 women and 159 men) aged 19–25 (mean age: 22.6) were studied. The age of the women ranged from 19 to 24 (mean age: 22.4) and of the men—from 19 to 25 (mean age: 22.8). In order to examine the intensity of transgression, the Polish version of the chronic self-destructiveness scale was applied. The gender was studied by means of the Polish version of the Bem sex role inventory. Androgynous women achieved the highest and feminine men—the lowest scores on the transgression scale. In women, the masculinity scale positively correlated with the transgression scale, whereas the femininity scale did not significantly correlate with transgression, although the coefficient was negative. No statistically significant correlations were found in men (although the coefficients were positive). Biological sex and gender were qualitative variables that differentiated the intensity of transgression. Equilibrium between the psychological dimension of femininity and the psychological dimension of masculinity was vital for transgressive tendencies, particularly in women. Androgynous women showed rather the adaptive aspect of transgression.

## Introduction

Biological sex and psychological gender are factors determining human activity. That activity may have consequences other than intended or completely unexpected, or even harmful to the individual, irrespective of the degree of awareness of the subject and regardless of the time perspective (now and immediately *vs.* later) or type of harm (physical *vs.* psychological harm).

Biological sex is a set of traits with which an individual is born; it seems to be an obvious and natural matter. In turn, psychological gender, and in particular the “configuration” of psychological feminine and masculine traits in every individual, independent of biological sex, seems to be less obvious and natural.

As the issue has been presented in many works [*cf.* 1], we shall limit ourselves to the most important statements in this paper. For a long time now biological structures, *e.g.* sex chromosomes, and psychological concepts such as gender identity have been distinguished; the term “sex” refers to physical traits of the individual and the term “gender” refers to psychological traits and behaviour of the human [2–4]. Sandra Lipsitz Bem rejected the traditional dichotomous or bipolar masculinity–femininity model deciding that people have both those traits with the higher or lower intensity independent of their biological sex. The configuration of psychological traits connected with gender (independent of biological sex) leads to four types of psychological gender. Sex-typed individuals possess psychological traits consistent with their biological sex (feminine women, masculine men). Androgynous individuals have to a great extent both feminine and masculine traits. Non-sex-typed (undifferentiated) individuals possess both feminine and masculine traits developed to a small degree. Cross-sex-typed (sex-reversed) individuals have psychological traits consistent with the sex opposite to their biological sex (masculine women, feminine men) [5–8].

Chronic self-destructiveness is described as a generalised tendency to undertake behaviours increasing the likelihood of negative and decreasing the likelihood of positive consequences for the subject [9]. For the purposes of this study, it was assumed that indirect/chronic self-destructiveness is behaviours whose likely negative consequence is intermediated by additional factors, while the relationship between the behaviour and harm is perceived as likely [10, 11]. There are, in general, several categories of indirectly self-destructive behaviours. A typical, or even textbook, example of indirectly self-destructive behaviours is transgression and risk-taking; it is such a manner of the individual’s behaviour that violates norms (*e.g.* school rules) and values generally accepted by the society, hence often the principles of community life. Such behaviours may also include, among others, gambling and risky behaviours undertaken for momentary pleasure, such as driving with bravado; transgressive behaviours also encompass illegal drug and alcohol use as well as smoking. That category also comprises succumbing to temptations, impulsiveness and seeking risky excitation [9–11]. Risk-taking may be of a destructive or adaptive nature. The destructiveness of risky behaviours is connected with the erroneous assessment of the gravity of danger and likelihood of its occurrence [10].

There are few studies into indirect self-destructiveness in general; there are even fewer studies into the gender differentiation of indirect self-destructiveness; and there are the fewest studies dedicated to the gender differentiation of transgressive tendencies and behaviours in a comprehensive, holistic manner [*cf*. 1, 12]. Most of the carried out research concerned direct self-destructiveness; it was found, for instance, that women exhibit passive self-destructiveness [13]. There were also studies into specific, isolated indirectly self-destructive behaviours (falling mainly into the category of risk) which indicated that men are more prone to such risky behaviours as abusing alcohol, not fastening seat belts in vehicles, performing hazardous work/occupations and criminal activity [14, 15]. In men, positive correlations between illegal drug use, aggressive or criminal activity, risky sexual behaviours, alcohol abuse and irresponsible behaviours as students or at work were found [16].

Many observations and research results prove that men display more transgressive behaviours, but most studies and data concern direct self-destructiveness; moreover, the world literature offers hardly any studies into relationships between transgressive tendencies and behaviours and types of psychological gender [*cf*. 1].

The aim of this study was to examine relationships between transgressive tendencies and behaviours and types of psychological gender.

## Methods

This study is part of research projects on indirect self-destructiveness and psychological gender, hence methods and some fragments may be similar to those of already published papers [*cf*. 1].

### Participants

A population of 558 individuals (399 women and 159 men) aged 19–25 (mean age: 22.6) was studied. The age of the women ranged from 19 to 24 (mean age: 22.4) and of the men—from 19 to 25 (mean age: 22.8). The study group was formed on the basis of the random selection from the general population (of healthy subjects); participation in the study was voluntary and anonymous. All the subjects were mentally and somatically healthy. The participants were heterosexual.

## Materials

In order to examine the intensity of transgression and risk in the study population, the transgression scale of the Polish version of the chronic self-destructiveness scale (CS-DS) by Kelley [9], as adapted by Suchańska [10], was applied. In order to examine chronic (indirect) self-destructiveness as a generalised tendency, Kelley created a research tool including four categories of behaviours; the final version comprises a set of 52 statements. Both the Polish and original versions of the tool are characterised by high reliability and validity [9, 10].

The psychological gender was studied by means of the Polish version of the Bem sex role inventory (BSRI) by Bem [5, 6], as adapted by Kuczyńska [7, 8]. Scores achieved for two dimensions (femininity and masculinity) enable to classify subjects as belonging to four types of psychological gender: sex-typed (masculine men, feminine women), androgynous (having feminine and masculine traits to an equal extent), cross-sex-typed (sex-reversed) (masculine women, feminine men), and non-sex-typed (undifferentiated) individuals. Both the original and Polish versions of the BSRI are characterised by high reliability and validity [5–8].

### Statistical Analysis

The statistical analysis of received results applied descriptive methods and statistical inference methods. In order to describe the mean value for quantitative traits, the arithmetic mean (M) was calculated, while the standard deviation (SD) was assumed to be the dispersion measure. The analysis of variance (ANOVA) and “post hoc” comparisons by means of the honestly significant difference (HSD) test by Tukey for unequal sample sizes were employed; in order to examine relationships between the studied variables, Pearson’s correlation coefficient r was applied. For all the analyses, the maximum allowable type I error was assumed at α = 0.05; *p* ≤ 0.05 was considered statistically significant. Statistical analyses were performed by means of the *Statistica PL 12.5 for Windows* [17] statistical package.

## Results

A recently carried out research project indicated that women achieved somewhat lower scores on the transgression scale than men, although the difference did not reach statistical significance [*cf*. 12].

Although transgression is among crucial indirect self-destructiveness categories and transgressive behaviours epitomise indirectly self-destructive behaviours, the distribution of scores on the transgression scale differed from the distribution of scores for indirect self-destructiveness as a generalised behavioural tendency [*cf*. 1].

In the study population, a majority of individuals were sex-typed (234 individuals, including 194 women and 40 men) and androgynous (196 individuals, including 127 women and 69 men); whereas cross-sex-typed individuals were the fewest (44 individuals, including 24 women and 20 men) and there were slightly more non-sex-typed individuals (84 individuals, including 54 women and 30 men).

**Fig. 1:**
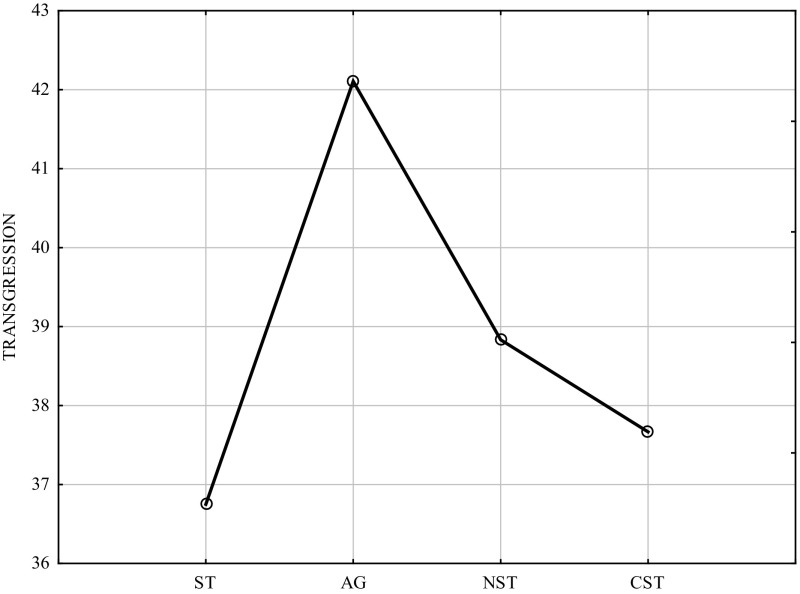
Subjects’ scores in the transgression scale of the CS-DS (psychological gender). Legend: *ST* sex-typed, *AG* androgynous; *NST* non-sex-typed (undifferentiated), *CST* cross-sex-typed

Table 1 and Fig. 1 indicate that the type of psychological gender (without taking into account biological sex) statistically significantly differentiated the intensity of transgression (ANOVA, F = 8.802; *p* = 0.00001; Tukey’s HSD for unequal sample sizes).

**Table 1:** ANOVA and post hoc comparisons of scores in the Transgression scale of the CS-DS (Tukey HSD for unequal N, 4 groups)

Independent (grouping) variable: Psychological gender	ANOVA, F = 8.802; *p* = 0.00001			
	ST M = 36.750	AG M = 42.107	NST M = 38.833	CST M = 37.667
ST		0.000001	ns.	ns.
AG	0.000001		0.02	0.01
NST	ns.	0.02		ns.
CST	ns.	0.01	ns.	

Legend: *ST* sex-typed, *AG* androgynous, *NST* non-sex-typed (undifferentiated), *CST* cross-sex-typed

The highest scores on the transgression and risk scale were achieved by androgynous individuals, while lower—by non-sex-typed, cross-sex-typed and sex-typed individuals (very similar scores). The lowest scores were obtained by sex-typed individuals; that result may be the converse of the above.

Non-sex-typed and cross-sex-typed individuals achieved scores similar to those of sex-typed individuals. Androgynous individuals most noticeably stood out.

A little more light can be shed on the above-presented results by examining differences in the intensity of transgression taking into account both psychological gender and biological sex.

**Fig. 2:**
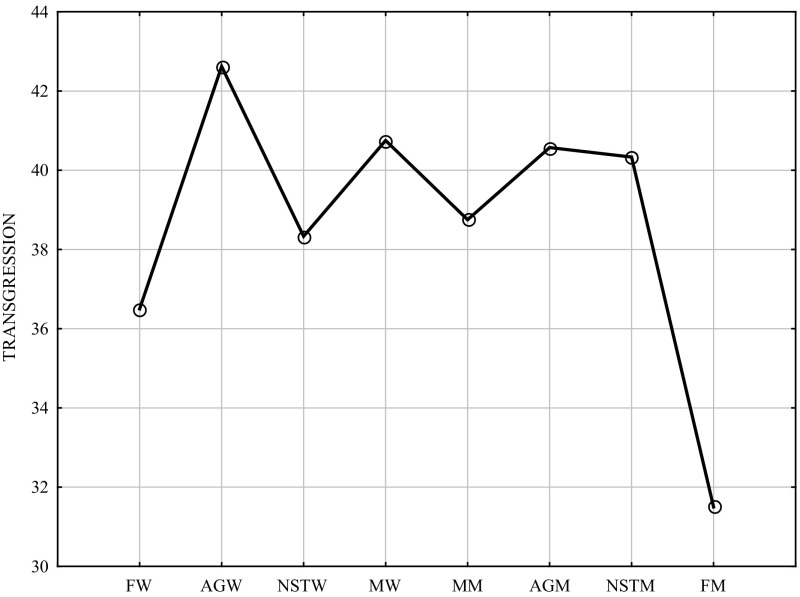
Subjects’ scores in the transgression scale of the CS-DS (biological sex and psychological gender). Legend: *FW* feminine women, *MW* masculine women, *MM* masculine men, *FM* feminine men, *AGW* androgynous women, *NSTW* non-sex-typed (undifferentiated) women, *AGM* androgynous men, *NSTM* non-sex-typed (undifferentiated) men

Table 2 and Fig. 2 show that biological sex and the type of psychological gender statistically significantly differentiated the intensity of transgression (ANOVA, F = 5.216; *p* = 0.00001; Tukey’s HSD for unequal sample sizes).

**Table 2:** ANOVA and post hoc comparisons of scores in the Transgression scale of the CS-DS (Tukey HSD for unequal N, 8 groups)

Independent (grouping) variables: (biological) sex, psychological gender; ANOVA, F = 5.216; *p* = 0.00001								
	FW M = 36.500	MW M = 40.750	MM M = 38.750	FM M = 31.500	AGW M = 42.619	NSTW M = 38.333	AGM M = 40.751	NSTM M = 40.333
FW		ns.	ns.	ns.	0.00000	ns.	0.01	ns.
MW	ns.		ns.	0.01	ns.	ns.	ns.	ns.
MM	ns.	ns.		0.04	ns.	ns.	ns.	ns.
FM	ns.	0.01	0.04		0.0003	0.03	0.006	0.02
AGW	0.00000	ns.	ns.	0.0003		0.009	ns.	ns.
NSTW	ns.	ns.	ns.	0.03	0.009		ns.	ns.
AGM	0.01	ns.	ns.	0.006	ns.	ns.		ns.
NSTM	ns.	ns.	ns.	0.02	ns.	ns.	ns.	

Legend: *FW* feminine women, *MW* masculine women, *MM* masculine men, *FM* feminine men, *AGW* androgynous women, *NSTW* non-sex-typed (undifferentiated) women, *AGM* androgynous men, *NSTM* non-sex-typed (undifferentiated) men

The highest score on the transgression and risk scale was achieved by androgynous women, followed by masculine women and androgynous men. The lowest score was obtained by feminine men; it is an interesting result which may become the subject of further research. The other types of psychological gender formed two clusters in respect of the scores on the transgression scale: scores of masculine women, androgynous men and non-sex-typed men were very similar (absence of sex-typed categories), whereas the other cluster encompassed feminine women, non-sex-typed women and masculine men (predominance of sex-typed categories).

The lowest scores were achieved by feminine men and feminine women.

Moreover, it can be seen that “extreme” scores were obtained by types with predominance of the psychological femininity dimension or absence of predominance of the psychological masculinity dimension (or at least equilibrium of the psychological femininity and masculinity dimensions): the highest score was achieved by androgynous women and the lowest—by feminine men and feminine women.

In order to examine relationships between transgression and specific types of psychological gender, a correlation analysis was performed (Pearson’s r).

In a recently published study, it was found that there were relationships between transgression and psychological gender dimensions: positive correlation with the psychological masculinity dimension and negative (although statistically non-significant) correlation with the psychological femininity dimension [1, 12]. Furthermore, positive correlation was observed between the masculinity scale and indirect self-destructiveness in women, whereas negative correlation was found between indirect self-destructiveness and the femininity scale in men [1].

This study attempts to explore relationships between transgression and risk and the psychological masculinity and femininity dimensions, for women and men separately.

**Fig. 3:**
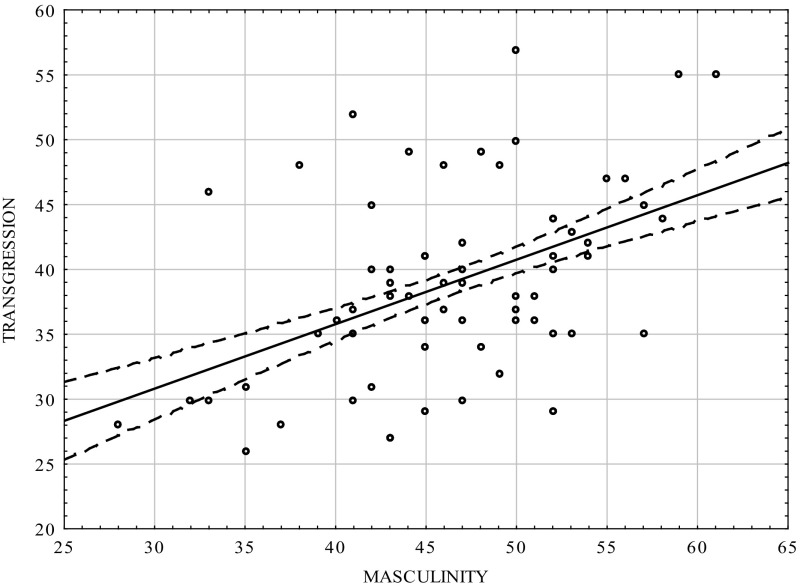
Scatterplot matrix of the scores in the transgression scale of the CS-DS and the Masculinity scale in the women group

**Fig. 4:**
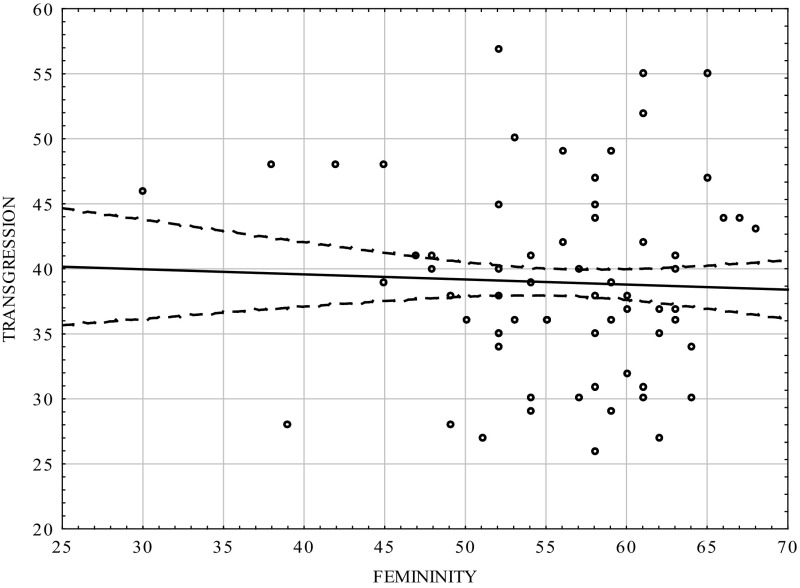
Scatterplot matrix of the scores in the transgression scale of the CS-DS and the Femininity scale in the women group

Table 3 and Figs. 3, 4 show that there was positive correlation between the masculinity scale and transgression scale in the group of women (0.464; *p* < 0.0001). The femininity scale did not statistically significantly correlate with transgression, but the sign of the coefficient was negative. The results were consistent with those for the whole population [*cf*. 12].

**Table 3:** Correlation coefficients between transgression and the dimensions of psychological gender in the women group

Variables	Masculinity	Femininity
Transgression	0.464	−0.03
	*p* = 0.0000000001	ns.

**Fig. 5:**
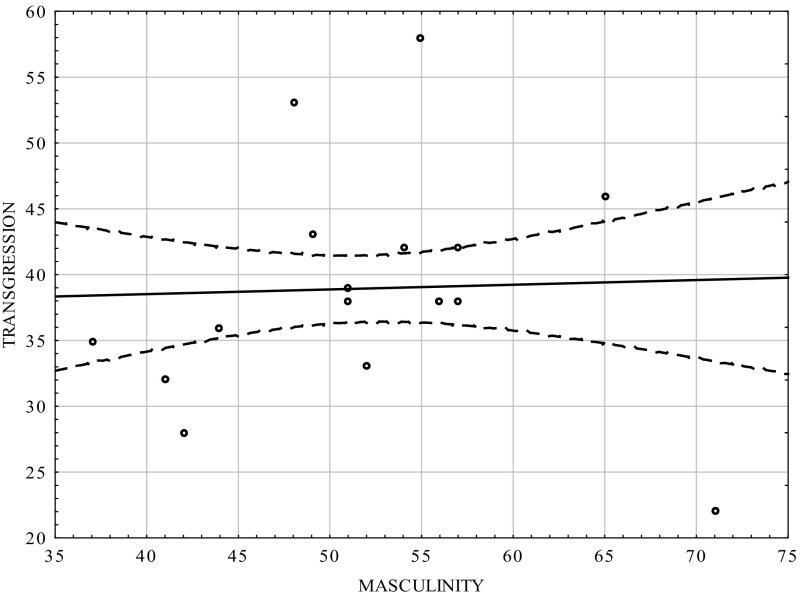
Scatterplot matrix of the scores in the transgression scale of the CS-DS and the Masculinity scale in the men group

**Fig. 6:**
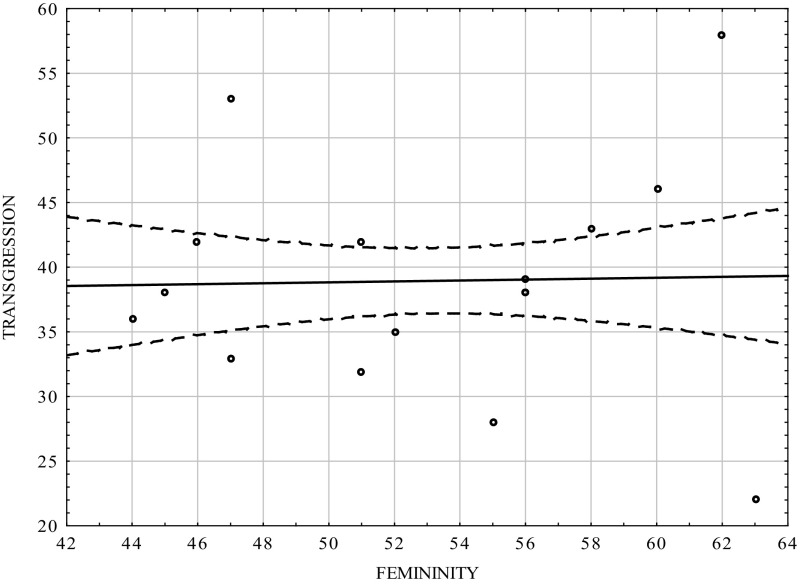
Scatterplot matrix of the scores in the transgression scale of the CS-DS and the Femininity scale in the men group

In turn, Table 4 and Figs. 5, 6 show that there was no statistically significant correlation between the masculinity scale or femininity scale and transgression in the group of men, while both the coefficients bore the positive sign.

**Table 4:** Correlation coefficients between transgression and the dimensions of psychological gender in the men group

Variables	Masculinity	Femininity
Transgression	0.193	0.143
	ns.	ns.

## Discussion

In the whole study population, there was the greatest number of sex-typed (feminine women, masculine men) and androgynous individuals; there were fewer non-sex-typed individuals, and the fewest cross-sex-typed individuals (masculine women and feminine men).

Due to the lack of studies in that scope, it will be difficult to refer to results of other research. One of the few studies indicated that men display a slightly higher intensity of transgressive tendencies (although without statistical significance) and that the psychological masculinity dimension is characterised by considerably higher predispositions towards undertaking transgressive behaviours than the femininity dimension [12].

A closer look ought to be taken at the higher intensity of transgression in androgynous and non-sex-typed individuals, and its lower intensity in sex-typed and cross-sex-typed individuals. Initially, Bem considered the two latter groups (*i.e.* those who are currently referred to as androgynous and non-sex-typed or undifferentiated individuals) to be androgynous ones, most likely due to the lack of predominance of any of the psychological gender dimensions. It was only later, in consequence of her studies inspired by research by Spence et al. [18], that she discovered differences between those two groups.

In the light of the data, she accepted the classification proposed by Spence, simultaneously drawing attention to the fact that both the groups belong to the common category of individuals who formed their concepts of the self outside the social definitions of femininity and masculinity. In androgynous and non-sex-typed individuals, the structure of psychological traits associated with gender and the shape of the concept of the self form outside the socially defined sex patterns [5, 8]. Therefore, there is every likelihood that it is where the source of the higher intensity of transgressive tendencies lies in those groups.

On the other hand, the significantly highest intensity of transgression among all the groups occurred in androgynous individuals, *i.e.* those characterised by equilibrium of both the strongly developed dimensions: femininity and masculinity. As observed above, non-sex-typed individuals have both feminine and masculine traits developed to a small degree and thus their intensity of transgression is more similar to the other groups. The highest intensity of transgression in androgynous individuals is to some extent understandable, taking into account the fact that psychological androgyny is an example of going beyond barriers, norms or socially and culturally established boundaries: it is not the predominance of traits making up the psychological gender dimension consistent with biological sex but the equilibrium of traits forming the psychological femininity and masculinity dimensions.^1^ Actually, as the author of the concept and tool for examining psychological gender states herself, in androgynous individuals the structure of psychological traits associated with gender and the shape of the concept of the self form outside the socially defined sex patterns [5].

The lowest intensity of transgression in sex-typed individuals ought to be similarly interpreted. Those individuals perform roles and display behaviours socially and culturally associated with their biological sex or even assigned to that. In other words, those individuals do not exhibit a tendency to cross boundaries in that or maybe not only that scope. In the extreme form, that may correspond with equivocation or conservatism in the form of behaviours meeting social expectations. Besides, the strong identification of the subject with the social stereotype of performing roles associated with sex or gender is not always beneficial for psychological or social functioning [19]. Norms connected with gender roles are among the strongest social norms which people are taught and which they internalise in the socialisation process encompassing gender roles [20, 21].

The above-presented differences, relationships and other regularities may become clearer if considered in the light of results received by applying, as qualitative predictors, both psychological gender and biological sex.

Biological sex, taking into account the type of psychological gender, differentiated the intensity of transgression. The highest intensity of transgression characterised androgynous women. Given the fact that in the whole population (not considering biological sex) the highest intensity of transgression occurred in androgynous individuals, it can be assumed that it was women who formed such a picture and gave the tone to the distribution of scores. It is an interesting result, worthy of consideration. At this point, it should be reminded that the intensity of indirect self-destructiveness as a generalised behavioural tendency in androgynous women was not so high: it was within the lower range of average scores [1]. When trying to interpret such a result, it should be kept in mind that transgression and risk are less intense in women [12]. Thus, does that actually concern the self-destructive aspect of transgressive tendencies and behaviours or maybe something else? Risky behaviours are typical of that area. Some authors point out not only the usefulness of risky behaviours but also certain positive aspects of those: propensity for risk-taking may be, among others, a manifestation of special tolerance and efficacy of coping mechanisms in psychological stress conditions, hence indicating increased adaptive abilities rather than self-destructiveness [10, 22, 23]. The destructiveness of a risky behaviour is connected with the presence of a trans-situational “recklessness” pattern and mistakes made in the assessment of the gravity of dangers and likelihood of their occurrence [10].

However, taking into account the fact that women show lower propensity for self-destructive behaviours in general, it can be assumed that it mainly concerns the aspect of crossing boundaries rather than the aspect of strictly risky behaviours; perhaps the adaptive significance of propensity for transgression and risk can be seen here. Following that line of thought, it can be assumed that, in androgynous women, it is not the indirectly self-destructive aspect of “recklessness” but rather the adaptive side of transgression in the sense of going beyond boundaries and norms socially and culturally established for their biological sex. As a matter of fact, in the transgressive concept of the human, the human by nature displays a tendency towards transgression, *i.e.* crossing boundaries and going beyond what has hitherto been achieved [24, 25]; an even earlier study carries a similar overtone, that time in the area of cognitive processes psychology: beyond the information given [26]. The essence of both the concepts is an assumption that, based on the possessed traits, data or information, the human is capable of creating something new, hitherto unknown, *i.e.* of going beyond oneself.

Androgynous individuals had the greatest psychological resources in the form of life satisfaction, optimism, sense of self-efficacy and competence [27]. Bem’s (hypo)thesis that an equilibrium of feminine and masculine traits occurring in androgynous individuals is an optimal pattern for mental health may be accurate; according to her, the condition for the fully effective human functioning is the complete integration of his or her masculinity and femininity into a more balanced, fuller, genuinely androgynous personality [5, 28].

As mentioned above, the second highest score for the intensity of transgression was achieved by masculine women. Thus, a statement can be ventured that the boundary was crossed to get as far as to the opposite end of dimension or continuum of masculinity–femininity. The third highest score was obtained by androgynous men. It stems from the rank order based on the intensity of transgression (three highest ranks: androgynous women, masculine women, androgynous men) that the psychological gender dimension opposite to biological sex is a factor favouring transgressive tendencies: for women—the psychological masculinity dimension, and for men—the psychological femininity dimension; a similar relationship was found in the case of indirect self-destructiveness as a generalised behavioural tendency [1].

Two clusters were formed in respect of the intensity of transgressive tendencies. The first cluster comprised masculine women, androgynous men and non-sex-typed men. It is worth noticing that there were no sex-typed categories in it. Moreover, although men predominated in the cluster (as far as biological sex was concerned), those were not necessarily masculine men: there was no predominance of the psychological masculinity dimension. On the other hand, the psychological masculinity dimension predominated in the group of women (masculine women). The other cluster included feminine women, non-sex-typed women and masculine men. It was predominated by women (as far as biological sex was concerned) and sex-typed individuals (feminine women, masculine men).

As earlier observed, the lowest intensity of transgression occurred in feminine men and feminine women. That may mean that the psychological femininity dimension protects against transgression, as it does against indirect self-destructiveness as a generalised behavioural tendency [1, 12].

In the whole population, cross-sex-typed individuals achieved quite low scores; whereas the lowest scores among all the groups characterised feminine men (masculine women obtained relatively high scores). In the light of the above, it can be noted that it was thanks to feminine men that the scores were so low: feminine men affected such a distribution of results. That would suggest that the distinctive predominance of the psychological femininity dimension in men does not favour transgression as it also does not favour indirect self-destructiveness as a generalised behavioural tendency [*cf*. 1]. Possibly, it indeed concerns the self-destructive aspect of transgression as opposed to women, and the psychological femininity dimension protects men against the self-destructive aspect as it does against indirect self-destructiveness as a generalised behavioural tendency [*cf*. 1].

In the general population, transgression was positively associated with the psychological dimension of masculinity [12]. Similarly positive correlation between transgression and the masculinity scale in the group of women may indicate that the psychological masculinity dimension in women favours transgressive tendencies and behaviours. At this point, the issue of the self-destructiveness and adaptiveness of transgression in women should be raised again. As mentioned above, it is very likely that it is not necessarily the self-destructive aspect of transgression that occurs in that case and maybe it is actually the converse—the adaptive aspect?

In the general population, transgression negatively correlated with the psychological dimension of femininity (although without statistical significance) [12]. Similarly negative and statistically non-significant correlation occurred between transgression and the psychological femininity dimension in the group of women; in order not to draw unjustified conclusions (due to the lack of statistical significance), a hypothesis may only be put forward that the psychological femininity dimension seems to protect them against such behaviours or at least not to be of the considerable importance for transgression in women. It also stems from the above that the direction of relationships for the whole population was such due to or thanks to women, or anyway was affected by women, especially as there were no statistically significant correlations in the group of men. It is an interesting issue that may become the subject of further research.

Results of this study may prove useful in prophylactic and therapeutic work. It may be worth considering and taking advantage of the adaptive nature of transgressive tendencies and behaviours in psychological help or psychotherapy.

## Conclusions

Psychological gender was a factor that differentiated the intensity of transgression whose highest intensity occurred in androgynous individuals. Biological sex and psychological gender together were qualitative variables that differentiated the intensity of transgression: its highest intensity occurred in androgynous women and the lowest—in feminine men. The equilibrium between the psychological dimension of femininity and the psychological dimension of masculinity was vital for transgressive tendencies, particularly in women. The psychological dimension of masculinity was significant for the intensity of transgression in women. Androgynous women showed rather the adaptive aspect of transgression.

## Footnotes

1 Etymology–Transgression: An act that goes beyond generally accepted boundaries. In classical Latin, “*a going over, a going across*”, noun of action from *transgressus*, past participle of transgredi “*step across, step over; climb over, pass, go beyond*”, from *trans*- “*across*” + *gradi* (past participle gressus) “*to walk, go*”. (Barnhart R. (ed.) *The Barnhart Concise Dictionary of Etymology*, 1999, London, W & R Chambers; Webster’s Universal College Dictionary, New York: Gramercy Books, 1997; Harper D. Etymology Dictionary, 2015).
